# Riboswitch Detection Using Profile Hidden Markov Models

**DOI:** 10.1186/1471-2105-10-325

**Published:** 2009-10-08

**Authors:** Payal Singh, Pradipta Bandyopadhyay, Sudha Bhattacharya, A Krishnamachari, Supratim Sengupta

**Affiliations:** 1 Centre for Computational Biology and Bioinformatics, School of Information Technology, Jawaharlal Nehru University, New Delhi - 110067, India; 2 School of Environmental Sciences, Jawaharlal Nehru University, New Delhi - 110067, India

## Abstract

**Background:**

Riboswitches are a type of noncoding RNA that regulate gene expression by switching from one structural conformation to another on ligand binding. The various classes of riboswitches discovered so far are differentiated by the ligand, which on binding induces a conformational switch. Every class of riboswitch is characterized by an aptamer domain, which provides the site for ligand binding, and an expression platform that undergoes conformational change on ligand binding. The sequence and structure of the aptamer domain is highly conserved in riboswitches belonging to the same class. We propose a method for fast and accurate identification of riboswitches using profile Hidden Markov Models (pHMM). Our method exploits the high degree of sequence conservation that characterizes the aptamer domain.

**Results:**

Our method can detect riboswitches in genomic databases rapidly and accurately. Its sensitivity is comparable to the method based on the Covariance Model (CM). For six out of ten riboswitch classes, our method detects more than 99.5% of the candidates identified by the much slower CM method while being several hundred times faster. For three riboswitch classes, our method detects 97-99% of the candidates relative to the CM method. Our method works very well for those classes of riboswitches that are characterized by distinct and conserved sequence motifs.

**Conclusion:**

Riboswitches play a crucial role in controlling the expression of several prokaryotic genes involved in metabolism and transport processes. As more and more new classes of riboswitches are being discovered, it is important to understand the patterns of their intra and inter genomic distribution. Understanding such patterns will enable us to better understand the evolutionary history of these genetic regulatory elements. However, a complete picture of the distribution pattern of riboswitches will emerge only after accurate identification of riboswitches across genomes. We believe that the riboswitch detection method developed in this paper will aid in that process. The significant advantage in terms of speed, of our pHMM-based approach over the method based on CM allows us to scan entire databases (rather than 5'UTRs only) in a relatively short period of time in order to accurately identify riboswitch candidates.

## Background

Recent discoveries of noncoding RNAs (ncRNAs), RNA molecules that do not code for proteins but function directly, reveal that they are abundant, widespread and perform truly diverse functions. [1,2] Significant and rapid advancements in RNA-mediated genetic control studies have established the importance of RNA in gene regulation [3,4]. The catalytic and regulatory roles of RNAs like ribozymes and riboswitches lend support to the hypothesis of RNA world and highlight the importance of RNA in the primordial world [5,6].

Riboswitches are *cis*-acting regulatory RNAs residing in the 5' untranslated regions (UTRs) of primarily prokaryotic mRNAs. They are complex folded structures that act as high affinity receptors for specific cellular metabolites [7-9]. On metabolite binding they undergo conformational change, which modulates gene expression at post-transcriptional level, either through premature termination of transcription [10] or inhibition of translation initiation [11]. They are composed of two structural domains: an aptamer domain [12] and an expression platform [13]. The aptamer domain binds the metabolite with high specificity resulting in the alteration of the RNA folding pattern mainly in the expression platform. Switching between two alternative RNA conformations, one of which is favoured in the absence of the bound metabolite and the other in its presence, leads to regulation of gene expression. The aptamer domain is highly conserved both at sequence as well as structure level among widely divergent organisms whereas the expression platform is highly variable even amongst the same riboswitch class. Riboswitches regulate genes in several metabolic pathways involved in the biosynthesis of vitamins, amino acids and purines [14,15].

Riboswitches have various important applications. Since they are believed to be the descendants of ancient metabolite sensors, they can be useful in gaining valuable insights into how gene regulation mechanisms evolved from the primitive forms of life to the more complex ones. Riboswitches have also been used as potential drug targets for antibacterial and antifungal agents [16]. Examples of such antimicrobial drugs are Pyrithiamine, which targets the TPP riboswitch [17] and S-(2-aminoethyl)-L-cysteine (AEC) which acts by binding to the lysine riboswitch [18]. Artificial riboswitches have also been engineered for the manipulation of gene expression; for example a theophylline-sensing synthetic RNA switch causes reduced access to an adjacent Shine Dalgarno sequence on theophylline addition [19]. Elucidating the underlying principles of riboswitch-mediated regulation may lead to the development of engineered ligands capable of modulating gene expression. More detailed characterization of the distribution and function of riboswitches across and within different genomes is essential to determine their precise role as riboregulators and potential drug targets.

Enormous growth of genome sequence data makes it practically infeasible to discover riboswitches solely by experimental means. In order to understand the extent to which organisms use these regulatory RNAs, time efficient algorithms for genome wide identification of riboswitches are required. Algorithms for detecting RNA homologs can be divides into two classes, those which are specific to a particular RNA class (e.g. tRNAscan-SE, miRscan etc.) and those which are general approaches applicable to all structured RNAs (e.g. INFERNAL). Each approach has its advantages and disadvantages. The specific tools use family specific properties to maximize speed and sensitivity but a new approach is required for each new RNA class. General tools can be used to detect members of any RNA class; however they are slower.

The most sensitive general-purpose method available for riboswitch search is the Covariance Model (CM). CM can be viewed as profile stochastic context free grammar which scores a combination of sequence consensus and RNA secondary structure consensus. Searches using CM require high quality hand curated RNA sequence alignments along with covariation information. These searches are complicated due to the incorporation of two levels of information and therefore require a huge amount of computing time. The search time scales roughly with the cube of the query length, so it becomes practically infeasible to search databases using larger RNA models.

The aim of this study is to develop a fast and efficient method for riboswitch identification. We propose profile Hidden Markov Models (pHMMs) [20] for consensus modelling of riboswitch sequences and their applicability for riboswitch detection. The method was used to search the Refseq database for riboswitches belonging to different classes. The whole genome search results as well as computational time required for the searches were compared with the Covariance Model. We find that our pHMM-based method is able to detect riboswitches belonging to eight of the ten families with high sensitivity and specificity while being more than a hundred times faster than the CM. We also compared our method with other web-based tools available for riboswitch discovery such as RibEx and Riboswitch finder. In both cases, our method is either more sensitive or as sensitive as the other method in detecting riboswitches. Our results indicate that pHMMs provide a fast and effective alternative for genome wide riboswitch searches.

## Results and Discussion

Hidden Markov Models (HMMs) [21,22] provide a coherent theory for probabilistic modelling of proteins and nucleotide sequences. HMMs have been demonstrated to be effective in detecting conserved patterns in multiple sequences [23]. A profile HMM (pHMM) [20,24] is an HMM with a structure that allows insertions and deletions in the model, and models gaps in a position dependent manner to give position sensitive gap scores. pHMMs can be constructed from a set of sequences belonging to a family and can be used for selective and sensitive database search for finding other members of that family. In this study we used two well known pHMM packages, *SAM *(referred in the text in uppercase italics to distinguish it from the Sam riboswitch family) [25] and HMMER [26] to construct pHMMs for each riboswitch family and used them to search for riboswitches in the Refseq database. *SAM *is known to be sensitive at model estimation while HMMER is known for more accurate model scoring [27]. Therefore *SAM *was used for pHMM construction and HMMER was used for database searching (as described in "Methods").

### Performance evaluation of the models constructed for different riboswitch families

**Figure 1:**
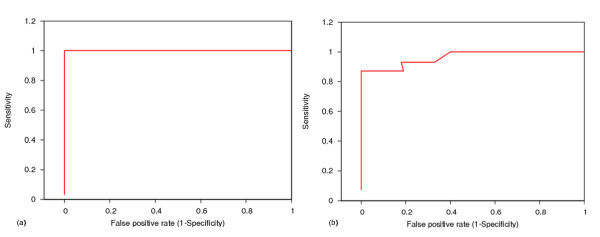
**Receiver-Operator Characteristic curves (ROC)**. (a) Lysine. (b) Sam alpha.

The pHMMs constructed for each riboswitch family were used to screen and classify the sequences in the test data set. The construction of the test dataset is described in Methods. For a given threshold score, a particular family model can classify member of a family in the test set either as a true positive (TP), if it correctly identifies it or as false negative (FN) if identifies it incorrectly as non family member. Similarly the model can classify non family members in the test set either as true negatives (TN), if predicts them correctly as non family member or as false positives (FP), if it predicts them incorrectly as a family member. Using these terms, sensitivity i.e. the fraction of the true matches that are accurately predicted by the method (calculated as TP/(TP+FN)) and specificity i.e. the fraction of all sequences predicted as matches that are indeed true matches (calculated as TP/(TP+FP)) may be used to measure the performance of a classifier. Receiver-Operator Characteristic (ROC) curves (1 - specificity v/s sensitivity)[28] generated for each family model indicate the discriminating potential of the HMM profile is high for all families except Sam alpha and PreQ1. ROC for two of the families Lysine and Sam alpha, are shown in Figure 1 and for other families the curves are provided in Additional files 1, 2, 3, 4, 5, 6, 7, and 8.

pHMMs for all the families except Sam alpha and PreQ1, show high sensitivity and specificity at the default HMMER threshold (threshold values for all classes are listed in the file "cutoff" in ). Sam alpha models were least sensitive while PreQ1 models were least specific. The cutoff threshold for PreQ1 was redefined so as to enhance the specificity of the model. The new threshold value of 7, at which PreQ1 model maintains high sensitivity and specificity, was selected. The threshold was decided on the basis of the scores assigned to the sequences in the test set. The sensitivity and specificity for all the families are reported in Table 1.

**Table 1:** Sensitivity and Specificity for different riboswitch families.

**Family**	**Sensitivity**	**Specificity**
FMN	0.99	1
Cobalamin	0.99	1
TPP	0.99	1
Purine	1	1
Sam	1	1
Sam alpha	0.67	1
Glms	1	1
Glycine	1	1
Lysine	0.97	1
PreQ1	0.95	0.95

### Comparison of pHMMs with the Covariance Models

**Figure 2:**
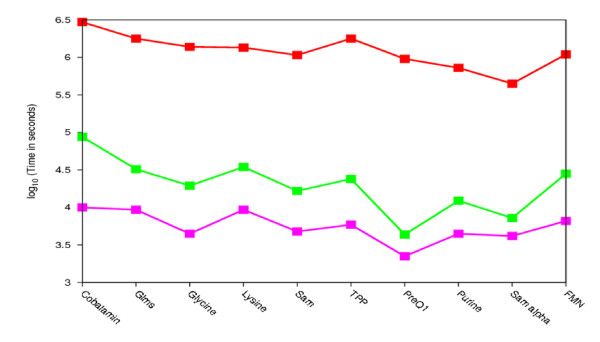
**Comparison of computational time of CM, RAVENNA and pHMM searches for different riboswitch families**. Red - CM, green - RAVENNA, pink - pHMM. Time is represented on log scale. It shows that pHMMs are several 100 times faster than CM. 73 complete genomes from Refseq database with size ranging from 20 KB to 13 MB were used to calculate computing time for different approaches.

Although models have been generated and evaluated on the constructed test set, however it may not necessarily reflect the true randomness and signals observed in real whole genome data. In order to test the performance of our method for genome wide searches, we used the pHMM models for scanning Refseq database [29] and compared the results with the current most successful general approach for ncRNA homolog detection, the Covariance Model (CM) [30]. CMs are probabilistic models that flexibly describe the secondary structure and the primary sequence consensus of an RNA sequence family. They are very sensitive and capture twice as much information as an HMM but have the drawback of being extremely slow. The main advantage of our pHMM-based method over the CM is the time factor. The pHMM-based method is several hundred times faster than the CM method. The scanning time (calculated over genomes of different lengths) taken by pHMM and CM for each riboswitch class is shown in Figure 2. pHMMs are extremely useful for large database searches as they are fast and take substantially less computing time.

CMs and pHMMs were used to scan the Refseq database for the candidates belonging to each of the ten riboswitch families. These families show different levels of sequence conservation and are of variable length. Some families like FMN and Sam are highly conserved while others like Cobalamin and Lysine show low sequence conservation.

**Figure 3:**
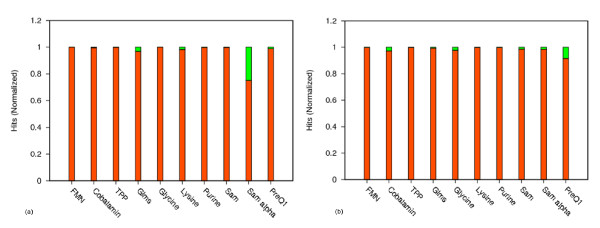
**Performance of pHMM for different riboswitch classes**. (a). Exclusive CM hits and common hits that are picked up by pHMM as well as CM are shown on normalized scale. Orange indicates common hits while green indicates hits picked exclusively by CM. (b). Exclusive pHMM hits and common hits that are picked up by pHMM as well as CM are shown on normalized scale. Orange indicates common hits while green indicates hits picked exclusively by pHMM.

The results obtained from the two different approaches were compared to determine how well the pHMM based models work for riboswitch identification. The genomic context of the exclusive hits were examined to determine their validity. While fool-proof validation of the exclusive hits can only come from their experimental detection, the relevance of the genomic context of the exclusive hits (the downstream genes are implicated in the ligand biosynthesis) allow for the possibility that these hits are genuine riboswitches. The performance of the pHMMs for each riboswitch class is shown in Figure 3. It was found that pHMMs work best for families characterized by distinct conserved sequence motifs in their aptamer region. Out of the ten riboswitch families studied here, eight families showed high CM hits coverage, ranging from 97.45% to 100% when hits upstream of hypothetical and putative genes are ignored and 96.83% to 99.95% when hits upstream of hypothetical and putative genes are taken into account. For two families, Sam alpha and PreQ1 the coverage was relatively low at 69.02% and 90.94% respectively. Very few sequences were available for model building in both of these cases. This suggested that the training set used for profile construction was inadequate to capture the full range of sequence variability within these families, thereby accounting for the poor performance of pHMM. Therefore models for these two families were built again from a larger training set. These training sequences were obtained after scanning Refseq database with pHMMs and filtering hits with E-vlaue less than 10e-5. However, models built from larger training sets also did not improve the results substantially for these two classes. It is known that both Sam alpha and PreQ1 have a very small aptamer domain with relatively simple secondary structure. Almost all riboswitch classes have highly conserved sequence patterns interspersed in their aptamer domains, which are modelled efficiently using pHMMs. However PreQ1 and Sam alpha being unusually small carry very few nucleotide positions that are highly conserved, thus making detection by the profile HMM method inadequate for these families. This explains the relatively low coverage of CM hits for these families. Thus for all families except Sam alpha and PreQ1, the two approaches were almost comparable. For all families, the exclusive CM and exclusive pHMM hits were analysed for their authenticity. The exclusive CM hits obtained for different families were found to be relatively low scoring thereby suggesting that these hits must have highly diverged sequence motifs which may not be detected just on the basis of sequence similarity using methods like pHMMs. The highest number of exclusive CM hits was found for Sam alpha. The exclusive pHMM hits obtained were also mostly low scoring and may be recovered using the CM by lowering the threshold score for identifying genuine riboswitches. However, this may increase the number of spurious hits as well. The highest number of exclusive pHMM hits was obtained for Cobalamin.

The overlap between CM and pHMM hits for each family is summarized in Table 2. Results for each riboswitch family are discussed in detail below.

**Table 2:** Percentage of CM hits covered by pHMM.

**Family**	**Total hits(CM)**	**Total hits(pHMM)**	**Common hits**	**% of CM hits covered by pHMM I**^**#**^	**% of CM hits covered by pHMM II**^**#**^
FMN	844	831	830	99.40%	100%
Cobalamin	1713	1807	1703	99.41%	99.59%
TPP	2245	2250	2242	99.95%	99.95%
Lysine	651	621	618	97.45%	98.25%
Glycine	1185	1260	1174	99.66%	100%
Purine	595	645	594	99.83%	99.83%
Sam	1262	1278	1255	99.44%	99.68%
Sam alpha	194	233	127	69.02%	75.14%
Glms	172	154	153	96.83%	97.45%
PreQ1	332	2387	267	90.94%	98.84%

I^# ^: percentage coverage was calculated after removing hits lying in gene, in AT repetitive region and those which were located far upstream of the genes. II^# ^: percentage coverage was calculated after removing hits lying in gene, in AT repetitive region, those which were far upstream of the genes, as well as those hits that were upstream of putative and hypothetical genes.

#### FMN riboswitch

This riboswitch class is characterized by the greatest degree of sequence conservation among members that are widely distributed across diverse bacterial species. When CM search results for FMN family were compared with that of pHMM, it was found that 99.40% of the CM search hits were obtained using pHMM based search. Exclusive CM and exclusive pHMM hits were analyzed in detail. When hits that are located upstream of hypothetical or putative genes were ignored, the percent hits covered by pHMM increased to 100%. Thus it is plausible that none of the exclusive CM hits appear be true positives. However one genuine hit was picked exclusively by pHMM.

#### Cobalamin riboswitch

This riboswitch class is also widely distributed amongst bacterial genomes. It has the largest average length and shows poor sequence conservation. A comparison of CM and pHMM results showed that 99.41% of the CM hits were reported by pHMM. After removing hits upstream to hypothetical and putative genes, this coverage increased to 99.59%. Seven genuine hits were found exclusively by CM search and forty-seven genuine riboswitch candidates were detected exclusively by pHMM search. The validity of the exclusive pHMM hits was determined by taking into account the genomic context in which they appeared.

#### Glms riboswitch

Glms is the only known riboswitch to exhibit ribozyme activity. It also shows high degree of sequence conservation and is found only in a few bacterial groups. For this family 96.83% of the total CM hits were also picked by the pHMM method. On closer inspection of the exclusive CM hits, it was found that many of these were in AT-rich repetitive regions that are unlikely to be valid riboswitches. Considering them as false positives and after excluding hits in the upstream of hypothetical and putative genes, only five genuine riboswitches were found exclusively by CM search and one genuine riboswitch candidate was found exclusively by the pHMM method.

#### Lysine riboswitch

The Lysine riboswitch shows low sequence conservation and is not very abundant in bacterial species. As in the case of the Glms riboswitch, many of the exclusive CM hits were in AT-rich repeat regions. After removing all such spurious hits, 97.45% of CM hits were recovered by pHMM search. When hits lying upstream to hypothetical and putative genes were discarded, only eleven exclusive CM hits and two exclusive pHMM hits were obtained.

#### Purine riboswitch

The Purine riboswitch is found in few bacterial groups and shows intermediate sequence conservation. For the Purine riboswitch 99.83% of the total CM hits were found using the pHMM model. One exclusive pHMM hit and one exclusive CM hit was found. There were no hits lying upstream to hypothetical or putative genes.

#### Sam riboswitch

The Sam riboswitch shows high-level sequence conservation. 99.44% of the total CM hits were recovered using the pHMM search method. After removing hits upstream to hypothetical as well as putative genes only three exclusive CM hits and seventeen exclusive pHMM hits were obtained.

#### TPP riboswitch

This is the most abundant riboswitch and is known to be present even in eukaryotes. It has intermediate level of sequence conservation. When CM hits were compared with those obtained using the pHMM method, it was found that 99.95% of the CM hits overlapped with the pHMM set. One exclusive CM hit and five exclusive pHMM hits were found to be true riboswitches on the basis of their genomic context. No hits upstream to hypothetical or putative genes were present in exclusive CM set.

#### PreQ1 riboswitch

PreQ1 has an unusually small aptamer domain with a simplified secondary structure consisting of a single stem loop structure. 90.94% of the CM hits were also obtained by pHMM search. After hits upstream of hypothetical and putative genes were eliminated, the coverage increased to 98.84%. However twenty four exclusive pHMM hits were found.

#### Glycine riboswitch

Glycine riboswitch is the only known metabolite binding riboswitch that consists of two metabolite binding aptamer domains in tandem. 99.66% of the CM search hits were obtained using the pHMM method. After discarding hits lying upstream to putative and hypothetical genes, twenty-seven exclusive pHMM hits were obtained; however no exclusive CM hits were detected.

#### Sam alpha riboswitch

The Sam alpha riboswitch is found predominantly in alpha proteobacteria. It is a short riboswitch with a relatively simple structure composed of a single hairpin. When CM hits were compared with the profile HMM results, it was found that the pHMM method covered only 69.09% of the CM hits. After discarding hits lying upstream to putative and hypothetical genes, forty-two exclusive CM hits were obtained and pHMM coverage of CM hits increases to 75.14%. Only two hits were detected exclusively by the pHMM method.

When we had nearly completed our analysis with covariance models using Infernal 0.72 [31], the new Infernal version 1.0 was released [32]. Since CM search requires a large amount of computing time, the new version implements two rounds of filtering to reduce the search time. The HMM filtering technique as described in [33,34] is applied first and then query-dependant banded CYK maximum likelihood search algorithm is used as a second filter [31]. It has been found that the default filters accelerate the similarity search by about 30-fold overall, while sacrificing a small amount of sensitivity. However, the models with little primary sequence conservation cannot be effectively accelerated by primary sequence based filters [32]. Although version 1.0 is faster than 0.72, it is still quite slow compared to pHMM searches. The comparison of riboswitch search times using Infernal 1.0 and our pHMM-based method, for different riboswitch families, is shown in the Additional file 9.

We also used Infernal 1.0 to scan the Refseq database for scanning the riboswitch families and found that at the same threshold (i.e. same as the one used for infernal 0.72 version), the hits reported by both the versions were similar except for TPP and PreQ1 where Infernal 0.72 reported more hits than Infernal 1.0. However Infernal 1.0 was found to be more specific as it did not report spurious hits in AT repetitive regions. Comparison of pHMMs with CM generated using Infernal 1.0 did not change the reported pHMM coverage of CM hits much (data not shown).

### Comparison with pHMM based heuristic for ncRNA detection

Extremely slow scans using CMs have inspired the use of heuristics to improve speed. Rfam uses a BLAST based heuristic. For each ncRNA family, the known members are BLASTed against RFAMSEQ; the full CM is run only on matches returned by BLAST. These searches are acceptably fast, but the BLAST heuristic may miss family members that would be found with a regular (slower) CM search. Profile HMM based filters such as rigorous filers and Maximum-Likelihood(ML) heuristics have also been developed [34,35]. Rigorous filters guarantee that all homologs detectable by a given CM are selected by the filter (i.e. ensures high sensitivity) but does so at the expense of speed since building rigorous filters can take several hours [35]. In ML-heuristic, profile HMMs are constructed from a given CM. The HMM transition and emission probabilities are designed to make the HMM maximally similar to CM [35]. These pHMM based filters have been implemented in the RAVENNA package. For each family CM, ML-heuristic profile HMM was built and used to scan the RefSeq database. The search speed was greatly enhanced as compared to CMs, nevertheless they were still slower (ranging from twice as slow to more than 10 times slower, depending on the riboswitch family) than purely sequence based profiles. The computational time required by an ML-heuristic profile HMM and sequence based pHMMs is compared in Figure 2. The number of hits obtained for most of the families (when an ML-heuristic profile HMM is used) is the same as that obtained from the CM searches. Therefore the percentage coverage statistics does not change.

### Comparison with other web based tools available for riboswitch identification

To determine the efficacy of our method relative to other riboswitch detection methods, we carried out a comparison of our approach with the Riboswitch finder and RibEx packages.

RibEx (Riboswitch Explorer) [36] scans RNA sequences for Riboswitch like elements (RLE) by examining its comprehensive list of overrepresented riboswitch sequence motifs [37] which has been compiled using the motif discovery and searching tools MEME [38] and MAST [39] respectively. Since MEME represents motifs as position-dependent letter-probability matrices that do not contain gaps, such an approach is likely to fail when functionally similar sequences show insertions or deletions within motifs. It is known that for the most abundant riboswitches, RibEx perform very well when compared with the co-variance models (~90% coverage when analysing bacterial sequences). However less common riboswitches (e.g. lysine and purine) are more difficult to model with sequence-based weight-matrices and RibEx recover between 70 and 80% of these riboswitch family members given in Rfam [36]. Also RibEx does not provide an option to search for PreQ1 and Sam alpha riboswitch family members. Since RibEx also follow a purely sequence based approach, it may not be very effective in detecting riboswitches belonging to these families which are characterized by short and low complexity sequence motifs. The comparison between RibEx and our pHMM-based method was carried out for remaining eight families. The performance of RibEx with pHMM was compared on the test set containing full family members for each of the riboswitch class obtained from Rfam database. The results are summarized in Table 3. Results clearly indicate that pHMMs miss fewer true positives for all riboswitch classes as compared to RibEx and hence show better accuracy.

**Table 3:** Comparison of the performance of the RibEx package with pHMMs.

**Family**	**Number of sequences in the test set**	**Number of sequences predicted by RibEx**	**Number of sequences predicted by pHMM**
FMN	183	183	183
Cobalamin	306	302	305
TPP	496	465	495
Glycine	217	184	217
Lysine	112	82	111
Purine	122	107	122
Sam	298	298	298
Glms	44	44	44

Another tool available for riboswitch identification is Riboswitch finder [40]. It uses sequence patterns, secondary structure prediction and scoring functions for the detection of a riboswitch in a given sequence. However this software is specifically designed for the purine-sensing riboswitch only. Earlier Riboswitch finder has reported a total of 18 putative purine riboswitches in genomic sequences of *Bacillus anthracis, Bacillus cereus, Enterococcus faecalis*, *Lactobacillus plantarum, Bacillus stearothermophilus, Clostridium tetani, Listeria innocua *and *Vibrio parahaemolyticus*. We scanned these genomes with Purine specific pHMM model and not only recovered the hits reported by Riboswitch finder but also found two new hits, one in *Bacillus anthracis *and the other in *Bacillus cereus*. We also scanned full members of Purine riboswitch family available in Rfam using Riboswitch finder. Riboswitch finder could detect only 114 out of 122 sequences listed in Rfam. In contrast, our pHMM-based method detected all of them.

## Conclusion

Accurate identification of riboswitches across entire genomes of varying lengths is the first step towards analysing the patterns in their intra and inter-genomic distribution. The distribution patterns of riboswitches can reveal important information regarding their evolution. It is therefore imperative to develop a framework for rapid and efficient detection of riboswitches across diverse genomes. Riboswitches are different from other ncRNA's by virtue of their relatively longer lengths and distinctive folding patterns. This is often manifest in the high level of primary sequence conservation that is observed between riboswitches belonging to the same family. This aspect has been exploited in our method of riboswitch detection.

The strength of the pHMM based approach for riboswitch identification lies in its speed as well as its accuracy (for all except two families) in identifying riboswitches. The success of the pHMM based approach to riboswitch identification depends on several factors such as the degree of primary sequence conservation, the presence of distinct and easily distinguishable sequence motifs in the aptamer domain and the availability of sufficiently large number of training sequences for model building, which adequately capture the distinct features of each riboswitch class. If the training set is small but the primary sequence conservation is high with distinct and easily identifiable motifs then the effectiveness of the pHMMs in detecting riboswitches will be high as in the case of FMN, Glms and Purine. Even for families with overall low sequence conservation (such as Cobalamin and Lysine) but which carry short stretches of multiple distinct motifs, pHMM performs extremely well. However if a family lacks highly conserved sequence motifs or has low complexity motifs, then the performance of pHMM will be poor as in the case of Sam alpha and PreQ1. Therefore these riboswitch families, which are characterized by short aptamer domains, lacking highly conserved sequence motifs cannot be found with high sensitivity and specificity using this approach.

We believe that the riboswitch identification framework developed in this paper (see also  for resources related to this paper) will be useful in screening genomic sequences to accurately and rapidly identify not only riboswitches but any other class of RNA's that are relatively long and characterized by multiple distinct sequence motifs.

## Methods

**Figure 4:**
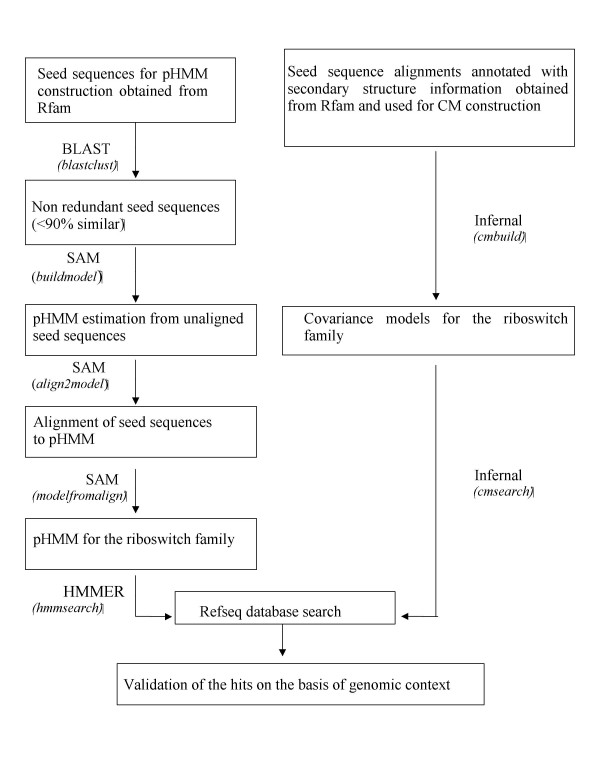
**Flowchart of the approach**. This figure illustrates the workflow of our approach

The workflow of our approach is illustrated in Figure 4.

### Training dataset for model building

Sequences for pHMM construction for each riboswitch family were obtained from the Rfam database (version 8.1) . Rfam is a comprehensive collection of ncRNA families, represented by multiple sequence alignments and profile stochastic context-free grammars (CM) [41]. "Seed sequences" which represent a set of known members of a riboswitch family were used to train the pHMM. It is necessary to remove redundant sequences from the training and testing data as it influences the performance of a method [42,43]. Therefore prior to model building, the training sequences were clustered on the basis of sequence similarity using *blastclust *[44]. Sequences that were 90% similar over 90% of their length were considered to be duplicates and hence were eliminated from the seed sequences thus generating the training set.

### Test dataset for model evaluation

The Rfam database was developed for the annotation of structured RNA families of genomic sequences, but it has been widely used as a source of reliable alignments and structures for the purposes of training as well as benchmarking RNA sequence and secondary structure analysis software. In order to test the performance of our method we obtained sequences from Rfam. For each RNA family Rfam provides seed sequences, which represent the known members of a particular family and the full collection of family sequences, which contains known members as well as those predicted by CM search. We downloaded fasta sequences of all the Rfam members filtered to less than 90% identity. This data not only includes the riboswitch family sequences but also contains over 597 other regulatory RNAs which have been compiled after scanning over 400 complete genomes. This data set was screened and the training sequences used for building pHMMs for each of the riboswitch family were removed. Fifty random sequences were also generated and included in the test set.

### Profile Hidden Markov Model construction

There are two packages available for pHMM construction, *SAM *and HMMER. The model was estimated using *SAM's *expectation maximization algorithm, *buildmodel*. The alignment of the training sequences to the resulting HMM was accomplished with *SAM's align2model *program. pHMMs were then constructed using the *modelfromalign *program which uses alignment generated by the *align2model *program. The profiles thus obtained were converted to HMMER-compatible format using the program *sam2hmmer *available with the *SAM *package. The profiles were then used to search microbial sequences in the RefSeq database version 28 using the *hmmsearch *program from HMMER. The pHMMs for different riboswitch families are provided in Additional files 10, 11, 12, 13, 14, 15, 16, 17, 18 and 19. The detailed commands and the pHMMs can be obtained at 

### CM model construction

In order to objectively compare the computing times of the pHMM and CM methods, it was necessary to carry out riboswitch searches using both methods on the same computing platform. Therefore, covariance models were constructed using the Infernal software package version 0.72 . CM describes both the secondary structure and the sequence consensus of an RNA. CM construction needs sequence alignment along with secondary structure annotation, therefore they were trained on the seed sequence alignments available in Rfam (version 8.1) using the *cmbuild *program from Infernal. These are manually adjusted alignments annotated with secondary structure information. CMs thus constructed were then used to search microbial genomes in the Refseq database using *cmsearch *program from Infernal. Rfam "gathering threshold" was taken as the cutoff threshold for each family (both for CMs as well as for ML-heuristic pHMMs). All the hits scoring above the threshold for the respective families were considered as legitimate riboswitch candidates.

### Calculating pHMM coverage of CM hits

The results of the pHMM and the CM searches were compared to obtain the sets of common hits picked by both the approaches and the hits picked exclusively either by the pHMM or the CM method. Known riboswitches are generally present at the 5'-ends (UTRs) of the genes implicated in the metabolism of their target molecules. Therefore, genomic contexts of the hits can be used to ascertain the authenticity of the riboswitches identified exclusively by either of the search methods. The exclusive hits obtained from both the approaches were examined with respect to the genomic context of the downstream gene to calculate the percentage of CM hits covered by the pHMM. The percentage was calculated in two different ways and is reported in Table 2. Hits located within the genes or far upstream of the genes (thousands of base pairs upstream) were considered as false positives. Hits lying in repetitive regions were ignored. Hence, the estimation of the percentage coverage of CM hits by pHMM hits was calculated after removing all the above mentioned false positives. For a conservative estimate we included the hits lying upstream of hypothetical or putative genes because such hits may possibly be indicative of genuine riboswitches. However, in the second case we calculated the percentage coverage by removing the hits upstream of hypothetical and putative genes also. In this case, only the hits upstream of genes known to be involved in the corresponding ligand biosynthesis pathway were considered to be legitimate candidates for calculation of percentage coverage.

## Authors' contributions

PS conceived and designed the study, wrote the programs, interpreted the data and wrote the manuscript. PB contributed to the design and development of the study, interpreted the data and helped draft the manuscript. SB contributed to the design and development of the study, interpreted the data and helped draft the manuscript. AK contributed to the design and development of the study and helped draft the manuscript. SS contributed to the design, development and coordination of the study, interpreted the data and wrote the manuscript. All authors read and approved the final manuscript.

## Supplementary Material

Additional file 1 **ROC curve for FMN riboswitch family**. PDF displaying ROC curve for FMN riboswitch family. Click here for file

Additional file 2 **ROC curve for Cobalamin riboswitch family**. PDF displaying ROC curve for Cobalamin riboswitch family. Click here for file

Additional file 3 **ROC curve for PreQ1 riboswitch family**. PDF description ROC curve for PreQ1 riboswitch family. Click here for file

Additional file 4 **ROC curve for Glms riboswitch family**. PDF displaying ROC curve for Glms riboswitch family. Click here for file

Additional file 5 **ROC curve for Glycine riboswitch family**. PDF displaying ROC curve for Glycine riboswitch family. Click here for file

Additional file 6 **ROC curve for Purine riboswitch family**. PDF displaying ROC curve for Purine riboswitch family. Click here for file

Additional file 7 **ROC curve for Sam riboswitch family**. PDF displaying ROC curve for Purine riboswitch family. Click here for file

Additional file 8 **ROC curve for TPP riboswitch family**. PDF displaying ROC curve for Purine riboswitch family. Click here for file

Additional file 9 **Computational time comparison of CM (1.0) and pHMM**. Blue - CM, Pink - pHMM. Time is represented on a log scale. It shows that pHMMs are several times faster than CM (1.0). 73 complete genomes from Refseq database with size ranging from 20 KB to 13 MB were used to calculate computation time for different approaches. Click here for file

Additional file 10 **profile Hidden Markov Model for FMN riboswitch family**. Text file displaying profile Hidden Markov Model for FMN riboswitch family. Click here for file

Additional file 11 **profile Hidden Markov Model for Cobalamin riboswitch family**. Text file displaying profile Hidden Markov Model for Cobalamin riboswitch family. Click here for file

Additional file 12 **profile Hidden Markov Model for PreQ1 riboswitch family**. Text file displaying profile Hidden Markov Model for PreQ1 riboswitch family. Click here for file

Additional file 13 **profile Hidden Markov Model for Glms riboswitch family**. Text file displaying profile Hidden Markov Model for Glms riboswitch family. Click here for file

Additional file 14 **profile Hidden Markov Model for Glycine riboswitch family**. Text file displaying profile Hidden Markov Model for Glycine riboswitch family. Click here for file

Additional file 15 **profile Hidden Markov Model for Purine riboswitch family**. Text file displaying profile Hidden Markov Model for Purine riboswitch family. Click here for file

Additional file 16 **profile Hidden Markov Model for Sam riboswitch family**. Text file displaying profile Hidden Markov Model for Sam riboswitch family. Click here for file

Additional file 17 **profile Hidden Markov Model for TPP riboswitch family**. Text file displaying profile Hidden Markov Model for TPP riboswitch family. Click here for file

Additional file 18 **profile Hidden Markov Model for Lysine riboswitch family**. Text file displaying profile Hidden Markov Model for Lysine riboswitch family. Click here for file

Additional file 19 **profile Hidden Markov Model for Sam alpha riboswitch family**. Text file displaying profile Hidden Markov Model for Sam alpha riboswitch family. Click here for file
