# Supplementary figures and images for: Riboswitch Detection Using Profile Hidden Markov Models
Source: BMC Bioinformatics. 2009 Oct 8;10:325. doi: 10.1186/1471-2105-10-325 (PMC2770071; doi:10.1186/1471-2105-10-325)

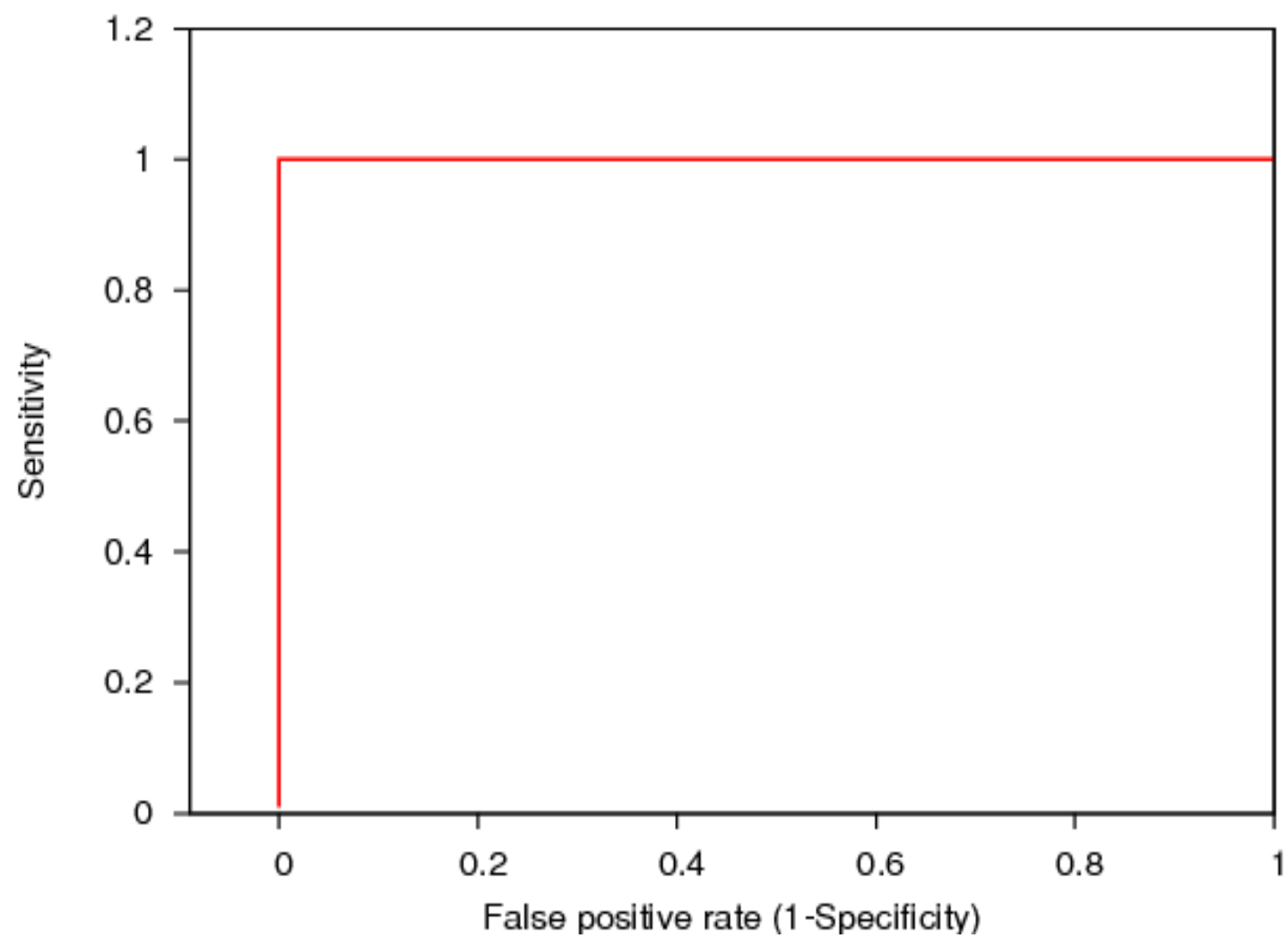

Supplement: Additional file 1 — ROC curve for FMN riboswitch family. PDF displaying ROC curve for FMN riboswitch family. [file 1471-2105-10-325-S1.PDF]

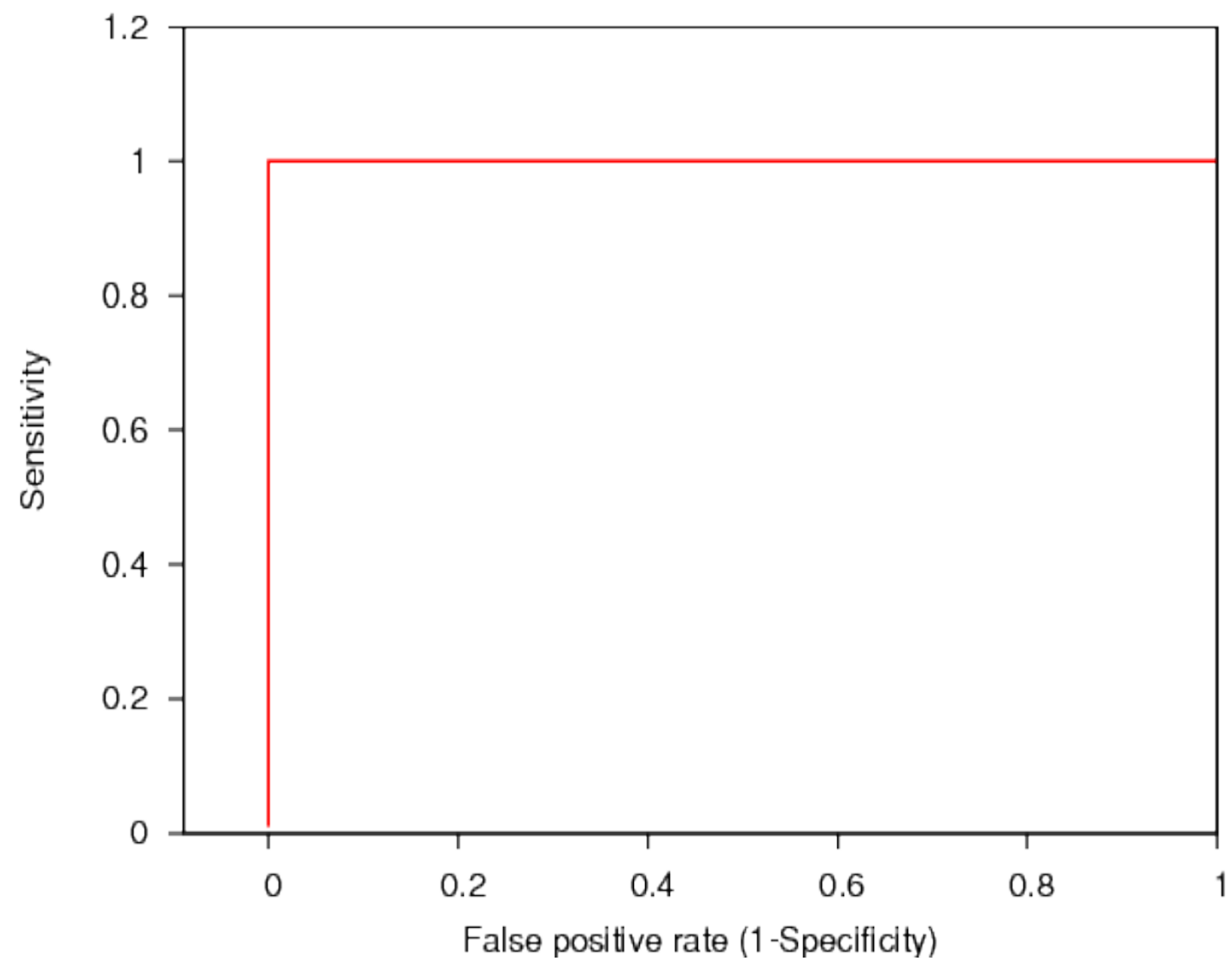

Supplement: Additional file 2 — ROC curve for Cobalamin riboswitch family. PDF displaying ROC curve for Cobalamin riboswitch family. [file 1471-2105-10-325-S2.PDF]

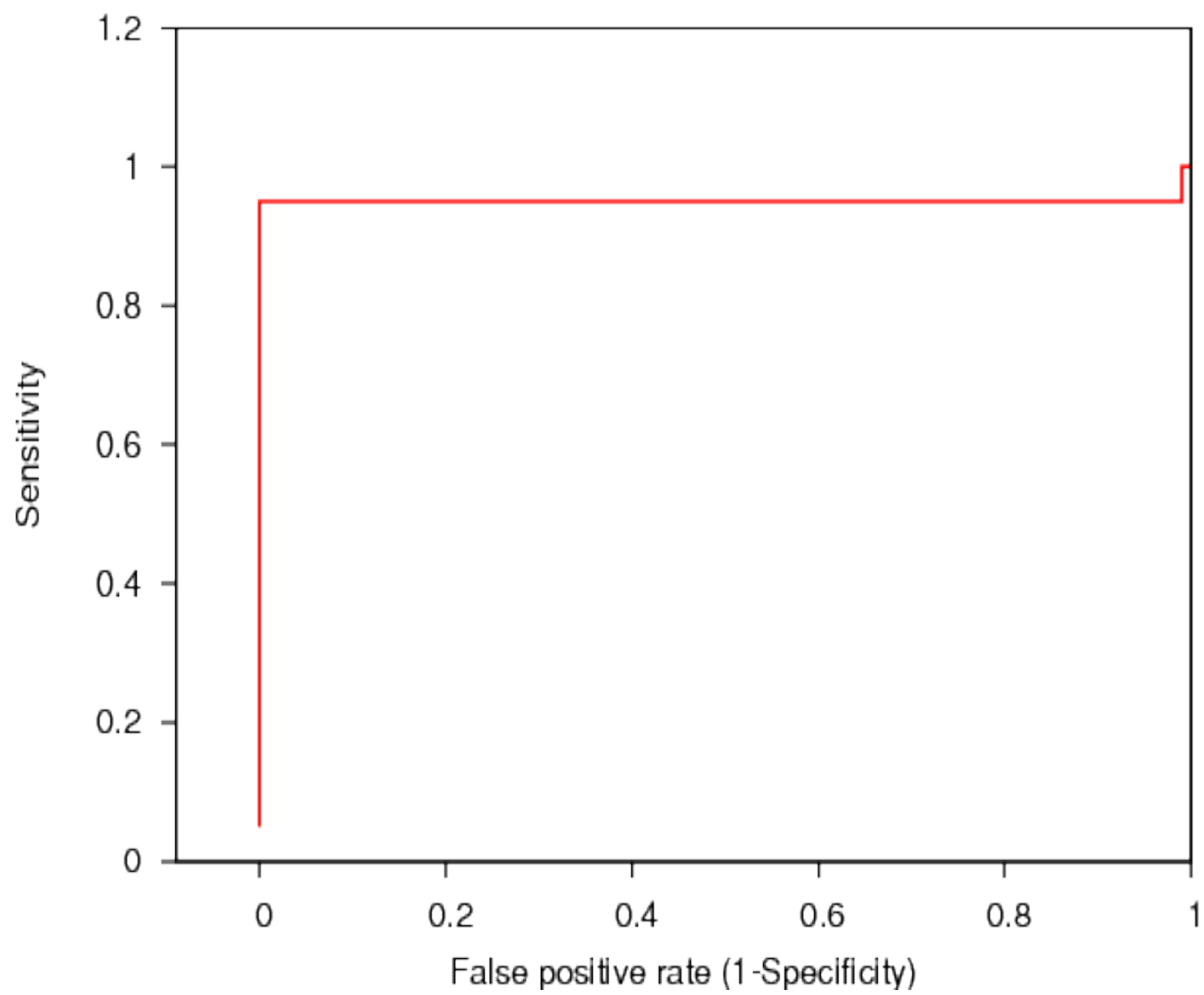

Supplement: Additional file 3 — ROC curve for PreQ1 riboswitch family. PDF description ROC curve for PreQ1 riboswitch family. [file 1471-2105-10-325-S3.PDF]

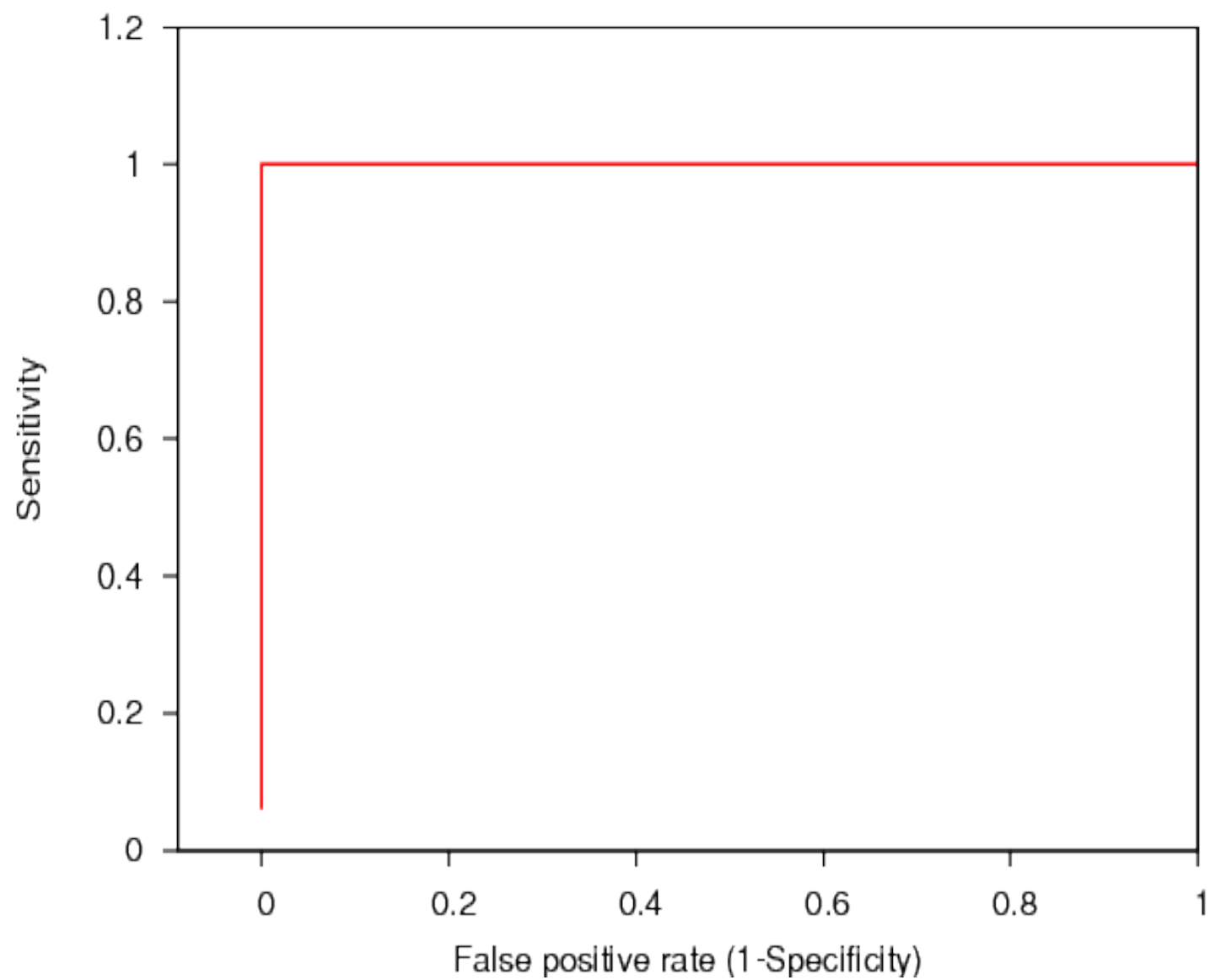

Supplement: Additional file 4 — ROC curve for Glms riboswitch family. PDF displaying ROC curve for Glms riboswitch family. [file 1471-2105-10-325-S4.PDF]

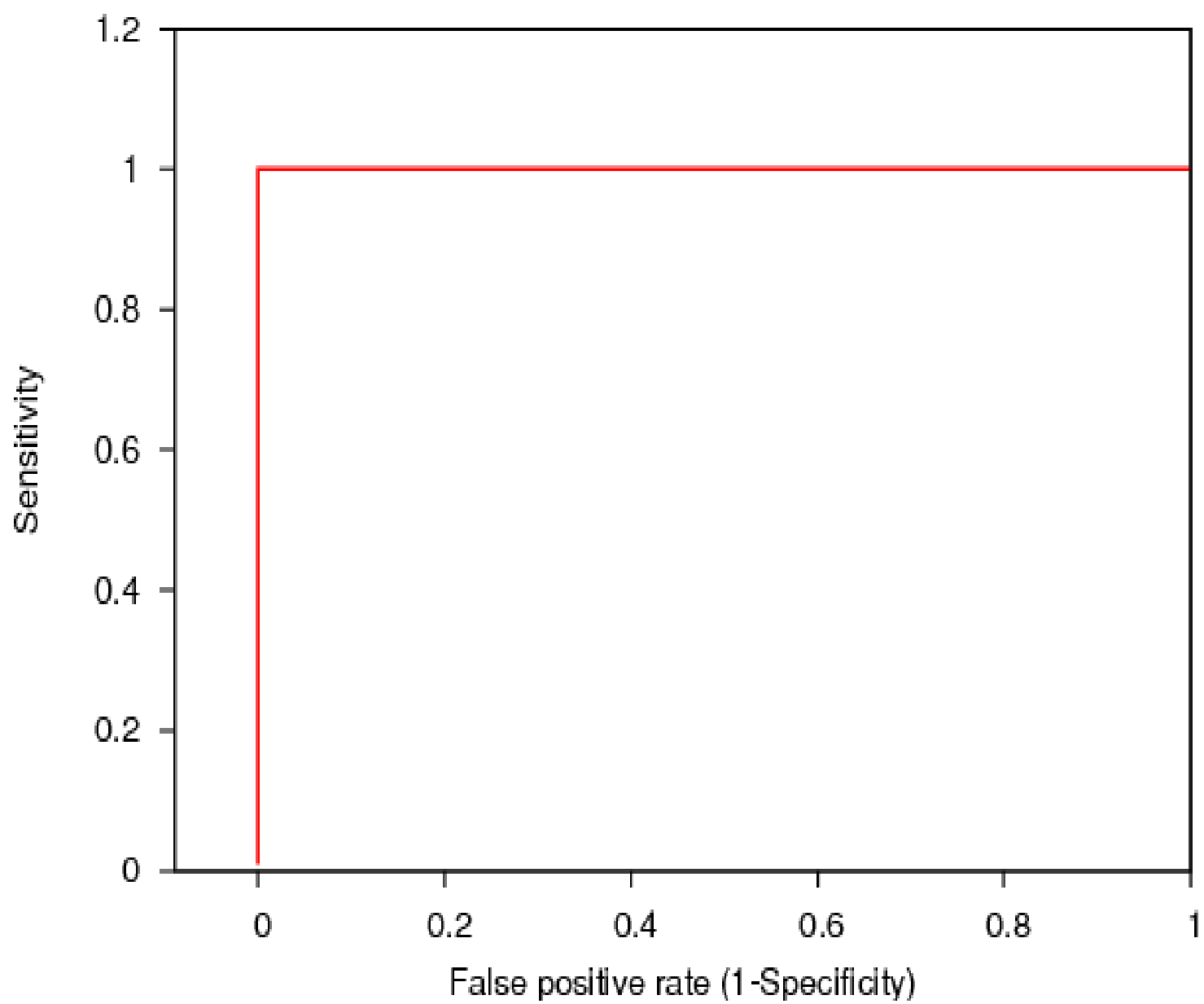

Supplement: Additional file 5 — ROC curve for Glycine riboswitch family. PDF displaying ROC curve for Glycine riboswitch family. [file 1471-2105-10-325-S5.PDF]

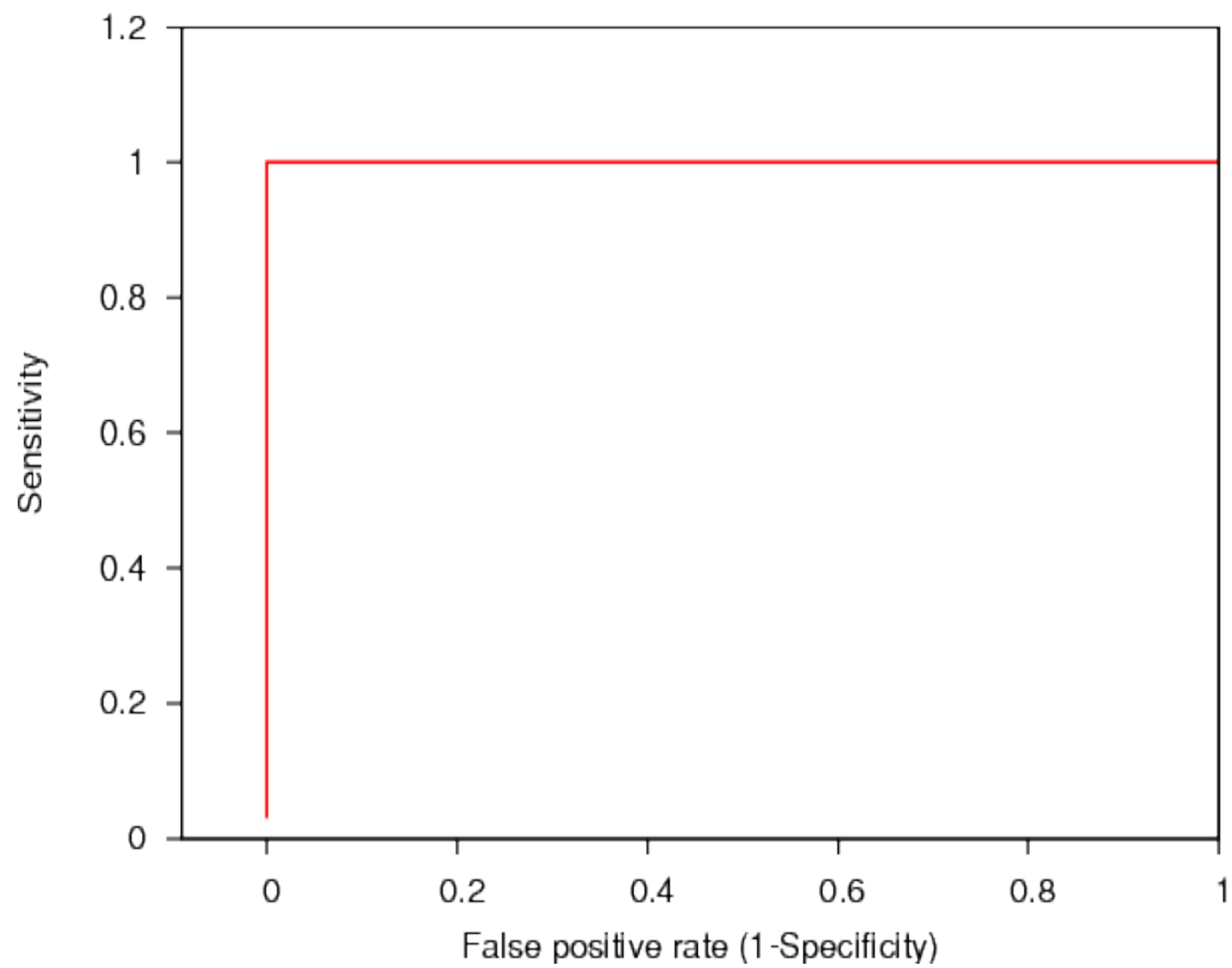

Supplement: Additional file 6 — ROC curve for Purine riboswitch family. PDF displaying ROC curve for Purine riboswitch family. [file 1471-2105-10-325-S6.PDF]

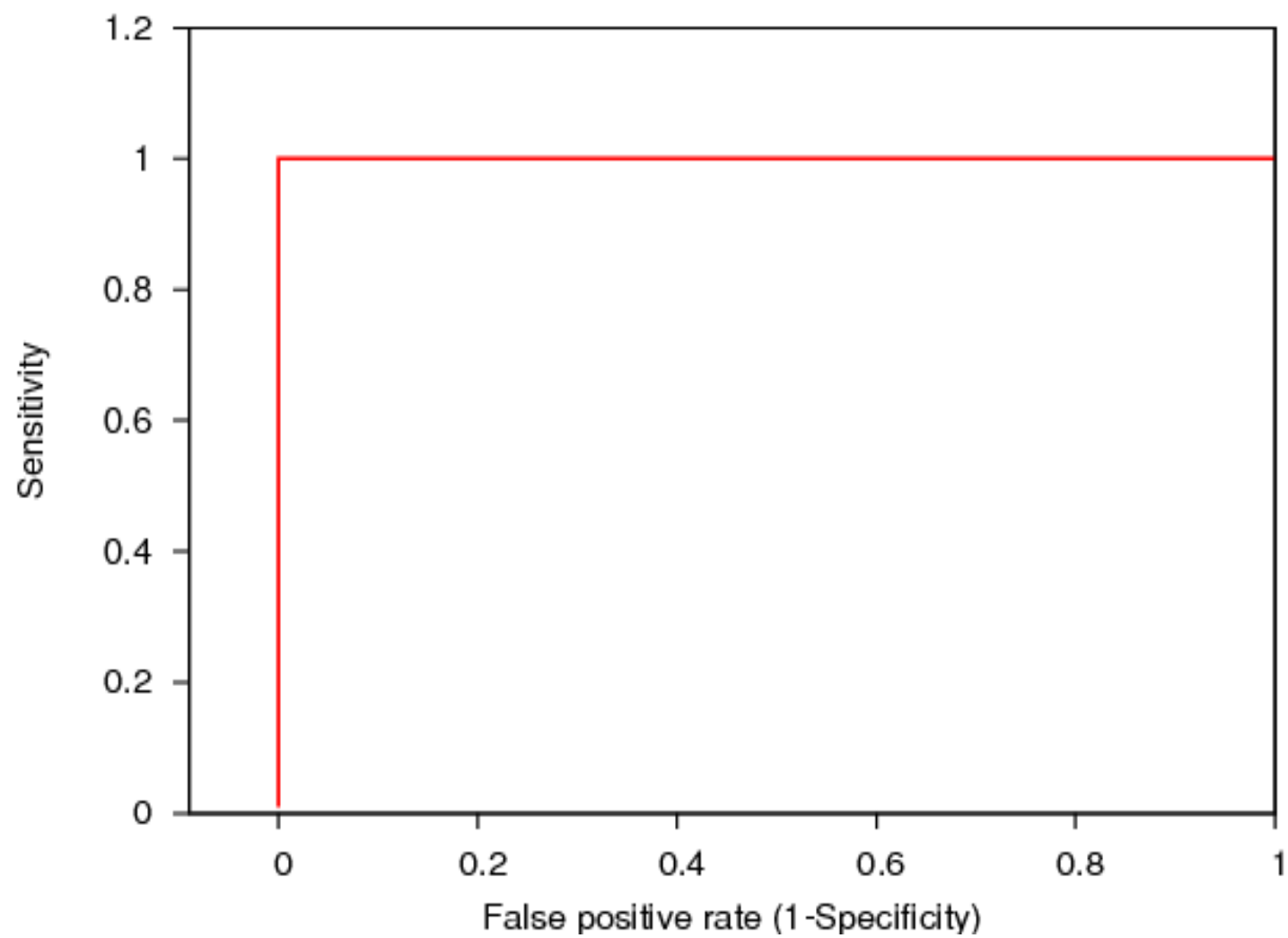

Supplement: Additional file 7 — ROC curve for Sam riboswitch family. PDF displaying ROC curve for Purine riboswitch family. [file 1471-2105-10-325-S7.PDF]

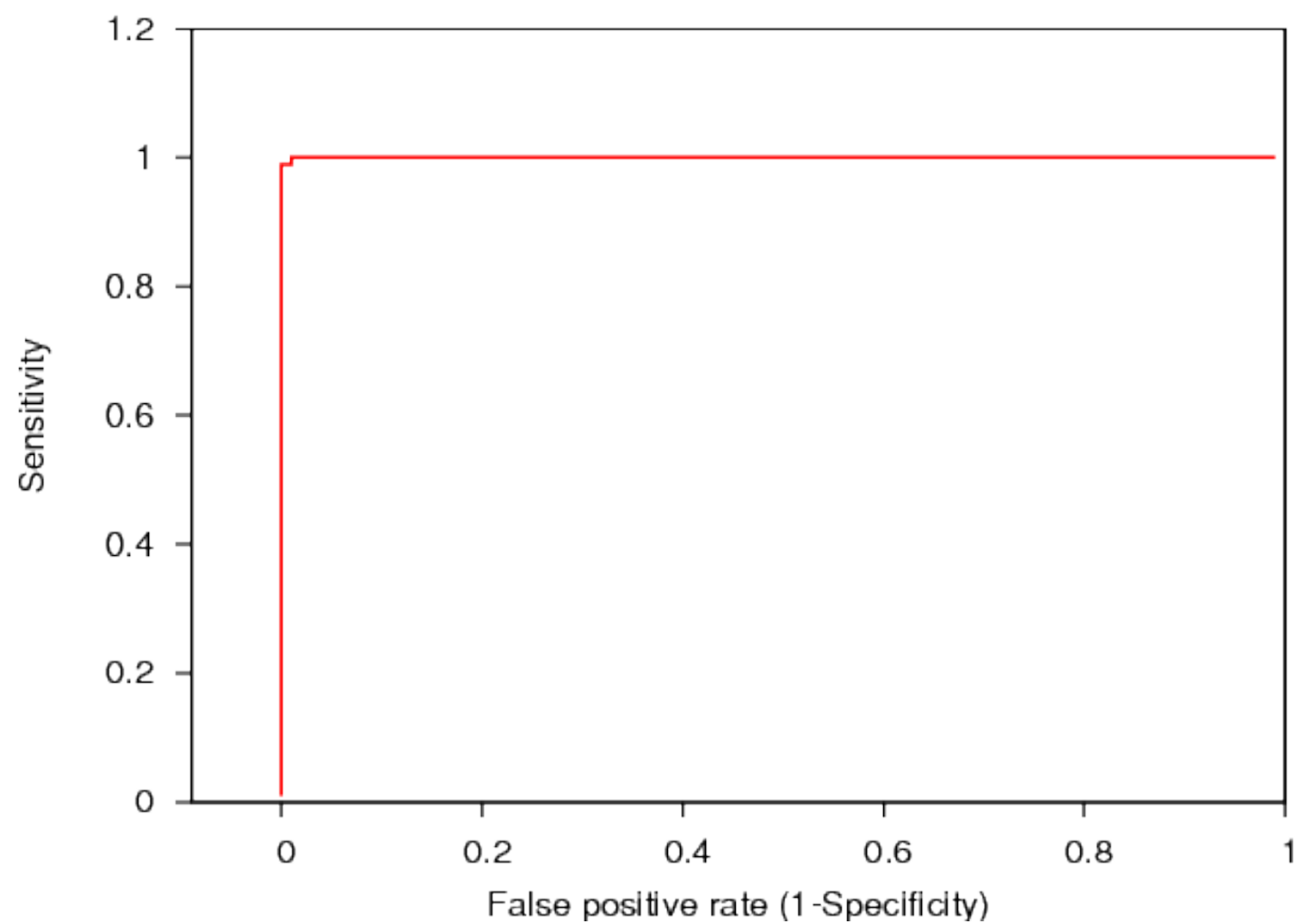

Supplement: Additional file 8 — ROC curve for TPP riboswitch family. PDF displaying ROC curve for Purine riboswitch family. [file 1471-2105-10-325-S8.PDF]

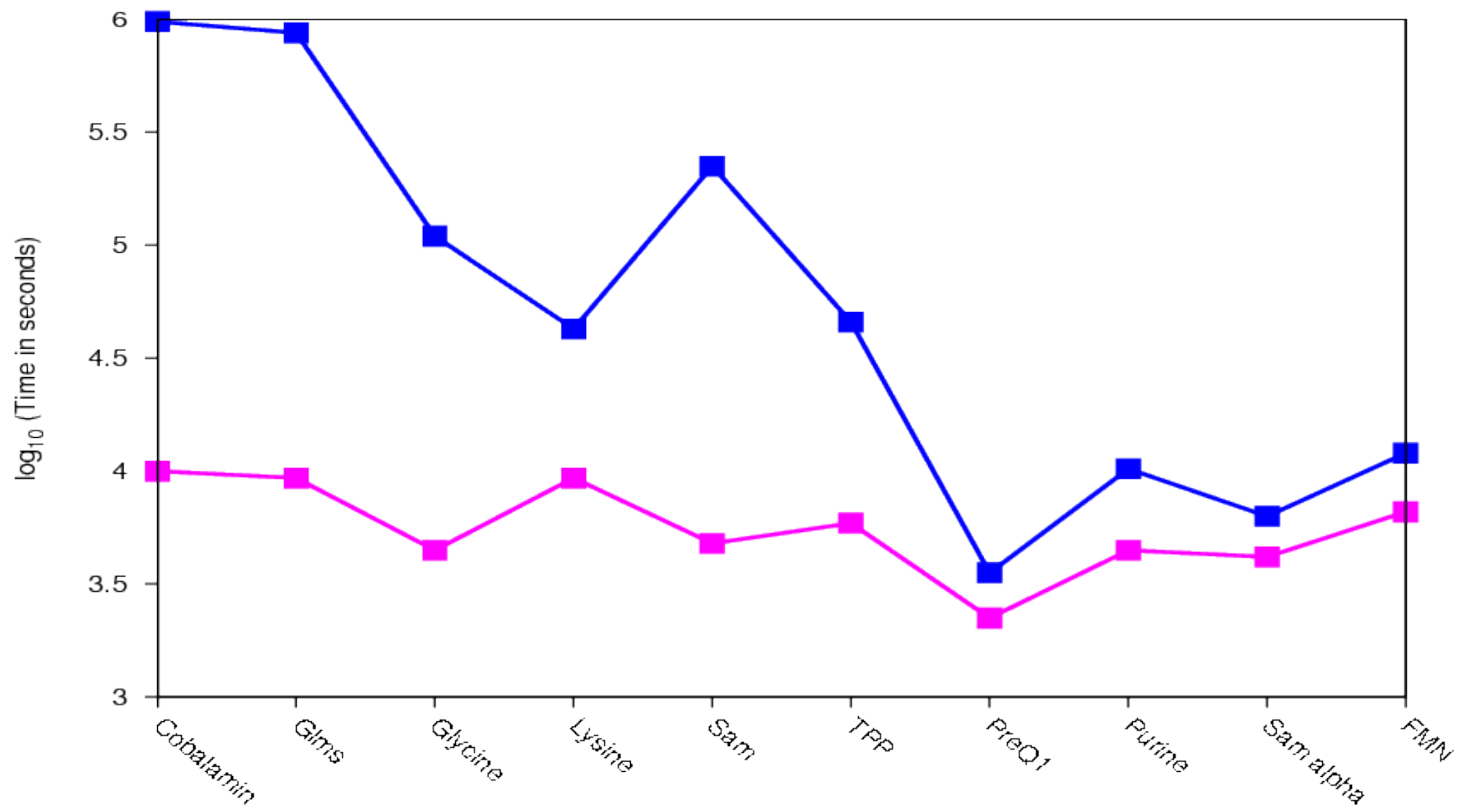

Supplement: Additional file 9 — Computational time comparison of CM (1.0) and pHMM. Blue - CM, Pink - pHMM. Time is represented on a log scale. It shows that pHMMs are several times faster than CM (1.0). 73 complete genomes from Refseq database with size ranging from 20 KB to 13 MB were used to calculate computation time for different approaches. [file 1471-2105-10-325-S9.PDF]
